# Raxibacumab augments hemodynamic support and improves outcomes during shock with *B. anthracis* edema toxin alone or together with lethal toxin in canines

**DOI:** 10.1186/s40635-015-0043-4

**Published:** 2015-02-28

**Authors:** Kenneth E Remy, Xizhong Cui, Yan Li, Junfeng Sun, Steven B Solomon, Yvonne Fitz, Amisha V Barochia, Mariam Al-Hamad, Mahtab Moayeri, Stephen H Leppla, Peter Q Eichacker

**Affiliations:** Critical Care Medicine Department, Clinical Center, National Institutes of Health, Bldg 10, Rm 2C145, Bethesda, MD 20892 USA; National Heart, Lung and Blood Institute, National Institutes of Health, Bethesda, MD USA; National Institute of Allergy and Infectious Disease, National Institutes of Health, Bethesda, MD USA

**Keywords:** *B. anthracis*, Edema toxin, Lethal toxin, Monoclonal antibody, Protective antigen, Raxibacumab

## Abstract

**Background:**

Lethal and edema toxin contribute to shock and lethality with *Bacillus anthracis*. We showed previously in a 96-h sedated canine model that raxibacumab, a monoclonal antibody against protective antigen, augmented hemodynamic support (HS) and improved survival with lethal toxin challenge. Here we study raxibacumab further. Using this model, we have now studied raxibacumab with 24 h edema toxin challenges (Study 1), and lethal and edema toxin challenges together (Study 2).

**Methods:**

Using our canine model, we have now studied raxibacumab with 24h edema toxin challenges (Study-1), and lethal and edema toxin challenges together (Study-2).

**Results:**

In Study 1, compared to no treatment, HS (titrated fluid and norepinephrine) increased mean arterial blood pressure (MAP, *p* ≤ 0.05) but not survival [0 of 10 (0/10) animals survived in each group] or median survival time [43.8 h (range 16.8 to 80.3) vs. 45.2 h (21.0 to 57.1)]. Compared to HS, HS with raxibacumab treatment at or 6 h after the beginning of edema toxin increased MAP and survival rate (6/7 and 7/8, respectively) and time [96.0 h (39.5 to 96.0) and 96.0 h (89.5 to 96.0), respectively]; (*p* ≤ 0.05). HS with raxibacumab at 12 h increased MAP (*p* ≤ 0.05) but not survival [1/5; 55.3 h (12.6 to 96.0)]. In Study-2, survival rate and time increased with HS and raxibacumab at 0 h (4/4) or 6 h after (3/3) beginning lethal and edema toxin compared to HS [0/5; 71.5 h (65 to 93)] (*p* = 0.01 averaged over raxibacumab groups).

**Conclusions:**

Raxibacumab augments HS and improves survival during shock with lethal and edema toxin.

**Electronic supplementary material:**

The online version of this article (doi:10.1186/s40635-015-0043-4) contains supplementary material, which is available to authorized users.

## Background

During recent outbreaks of *Bacillus anthracis* infection in developed countries, mortality rates in patients with shock have been close to 80% [1-4]. By comparison, mortality from shock due to other types of bacterial infection is reported to be 20 to 50% [5-8]. Adjunctive therapies that augment conventional hemodynamic support (HS) during *B. anthracis* shock may improve outcomes from this lethal infection.

*B. anthracis* produces two binary exotoxins, lethal (LT) and edema toxins (ET) [9-11], each consisting of a toxic moiety [lethal factor (LF) and edema factor (EF), respectively] and protective antigen (PA), which mediates transport of the toxic moieties into host cells. Lethal factor is a metalloprotease that blocks mitogen-activated protein kinase kinases and stimulates inflammasome formation [4,12,13]. Edema factor is a calmodulin-dependent adenylyl cyclase that increases intracellular cAMP levels [14-17]. Although on a weight basis LT is five to ten times more lethal than ET, both toxins are thought to contribute to shock during infection with *B. anthracis* [18-20].

We previously developed a 96 h sedated, instrumented, and ventilated canine model of *B. anthracis* toxin-associated shock in which LT or ET was infused over 24 h to simulate release during infection [18]. In this model, lethal doses of the two toxins had very different hemodynamic effects. While both caused hypotension that persisted for 96 h, LT produced myocardial dysfunction while ET produced marked arterial dilation. In this model, a non-lethal dose of ET augmented the lethal effects of LT [18].

We subsequently employed this model to investigate the treatment with hemodynamic support (titrated normal saline and norepinephrine infusions, HS) alone or together with raxibacumab (raxi), a PA-directed monoclonal antibody (originally Human Genome Sciences and now GlaxoSmithKline, Rockville, MD, USA) for LT-associated shock [21]. By targeting PA in the extracellular space, raxi inhibits the two toxins by blocking host cell internalization of the toxic moieties LF and EF, which is necessary for toxin activity [22-25]. Hemodynamic support alone produced a small but significant increase in survival [21]. However, compared to HS alone, HS with raxi administered either at the start of LT or 9 or 12 h later, promoted fluid mobilization, increased blood pressure, reduced vasopressor requirements, and improved myocardial function and survival [21]. Since raxi has now been added to the Strategic National Stockpile but has not been tested clinically during *B. anthracis*-associated shock, we employed this canine model to also investigate the effects of HS alone or together with raxi for shock caused by lethal ET challenge in one study (Study-1) and lethal challenge with LT combined with a similar weight dose of ET in another study (Study-2). We hypothesized that HS with raxi would improve survival and hemodynamics compared to HS in shock from ET alone or LT and ET together.

## Methods

### Study design

The National Institutes of Health Clinical Center Animal Care and Use Committee approved these studies as protocol CCM1201. Four purpose-bred beagles (weighing 10 to 12 kg) prepared as previously described with tracheostomy tubes and venous, pulmonary and systemic arterial, and urinary catheters, were studied weekly [18]. Sedation, maintenance fluids, and mechanical ventilation were applied similarly to all groups using standardized ICU protocols [21,26].

**Figure 1:**
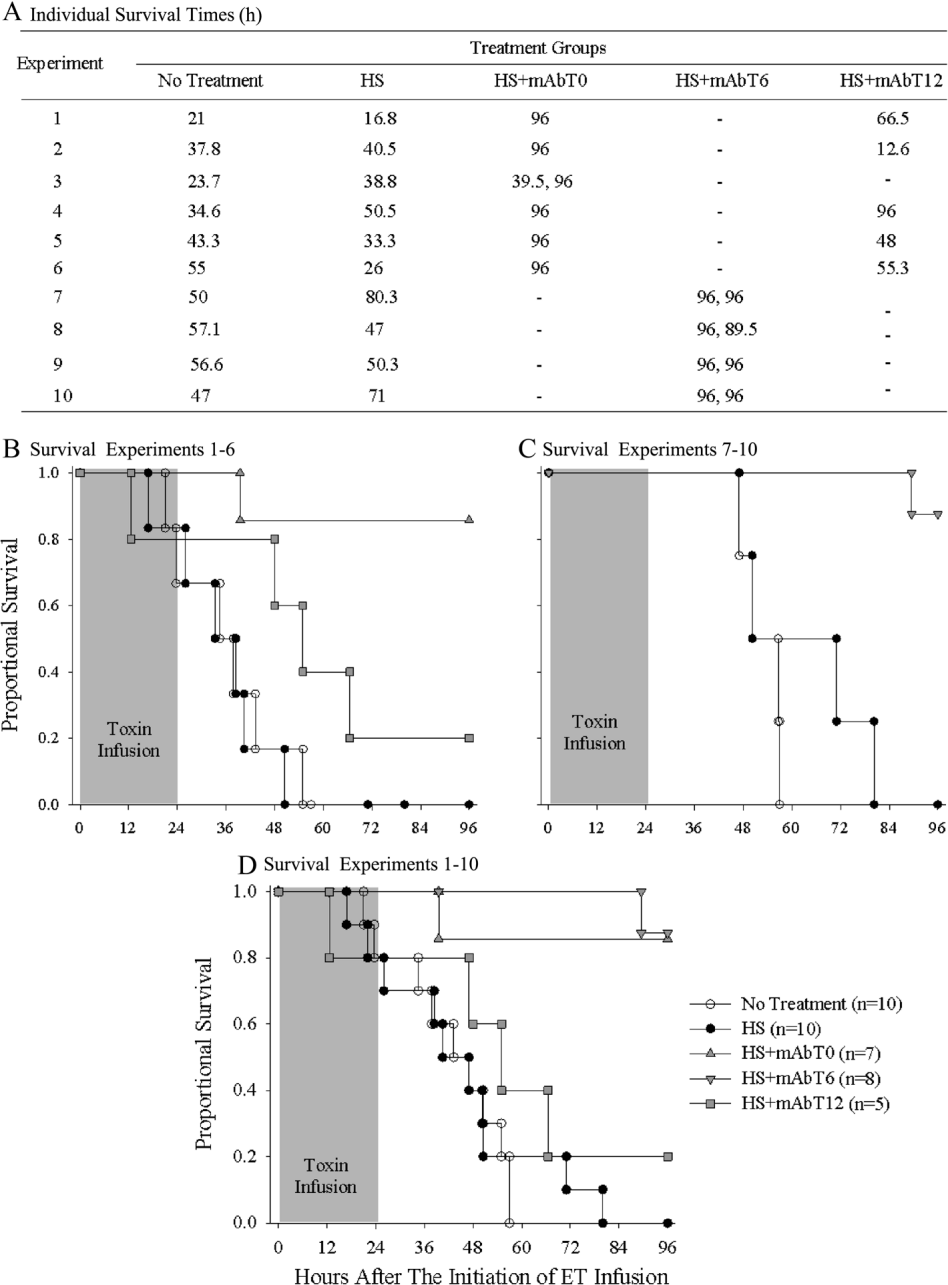
**Survival Times and the proportion of animals surviving over time.** This figure shows the individual survival times for each of the animals assigned to no treatment (maintenance fluid only), hemodynamic support alone (HS; fluids and norepinephrine titrated to pulmonary artery wedge pressure and mean arterial blood pressure, respectively), or HS combined with raxibacumab administered at the start of (HS + mAbT0) or 6 h (HS + mAbT6) or 12 h (HS + mAbT12) after the start of edema toxin (ET) infusion in each of the ten weekly experiments comprising Study 1 **(A)** (see Methods). This figure also shows the proportion of animals surviving over time in experiments 1 to 6 **(B)**, 7 to 10 **(C)**, and for all ten experiments together **(D)**. In experiment 3, an animal randomized to receive HS + mAbT12 received HS + mAbT0 instead.

In Study 1, starting at 0 h (T0), all animals were challenged with a 24 h ET infusion (Figure S1A in Additional file 1). Ten weekly experiments were conducted. Each week, in the first six experiments, four animals were randomized at the start of ET infusion to: no treatment (fixed maintenance fluid only), hemodynamic support alone (HS, fluid and norepinephrine titrated to pulmonary, and systemic arterial pressures, respectively), or HS combined with a single dose of raxi administered either at the start of ET (HS + mAbT0) or 12 h later (HS+ mAbT12) (Figure 1A). Based on the results of these six experiments (see Results), in the subsequent four experiments, each week four animals were randomized to no treatment (*n* = 1), HS alone (*n* = 1), or HS combined with raxi administered 6 h after the start of ET (HS + mAbT6) (*n* = 2).

In Study 2, four animals were challenged weekly with a lethal dose of LT combined with a similar weight dose of ET administered as 24 h infusions (Figure S1B in Additional file 1). Comparable doses of LT and ET together had previously additive effects on lethality compared to LT alone [18]. At the start of toxin infusion, animals were randomized to receive HS alone or HS combined with raxi administered at 0 h (HS + mAbT0) or 6 h (HS + mAbT6). As described in the results, after completion of three experiments, this study was concluded and data were analyzed.

Hemodynamic monitoring was started at 0 h and continued until the end of both Studies 1 and 2 (Figure S1A and B in Additional file 1). Cardiopulmonary and other laboratory measures were obtained immediately before and at regular intervals after initiation of toxin. Whereas blood pressure, heart rate (HR), temperature, and oxygen saturation were continuously monitored, central venous pressure (CVP) was recorded every 2 h, and pulmonary capillary wedge pressure (PCWP) was measured every 2 h for the first 8 h, and every 4 h thereafter. Arterial blood gases (ABGs) were obtained every 2 h for the first 8 h and every 8 h or as needed based on protocol thereafter. Left ventricular ejection fraction (LVEF), measured by echocardiography, and chemistry and complete blood count data were obtained at 24 h intervals. Total fluid intake was recorded every 2 h, whereas urine output was recorded every 24 h and at time of death. Norepinephrine requirements were recorded hourly. Surviving animals were euthanized at 96 h after measurements were completed.

### Toxin and treatments

Lethal and edema toxins were prepared as previously described and administered in doses designed to produce lethality rates greater than 75% [18]. In Study 1, the ET challenge consisted of PA, 410 μg/kg, and EF, 205 μg/kg; while in Study 2, LT and ET challenge together consisted of PA, 10 μg/kg, LF, 10 μg/kg, and EF, 10 μg/kg. The raxi dose employed in both studies (ten times the molar amount of PA administered to each animal) was administered as a single dose, 2 mL intravenous injection at the specified times (Figure S1 in Additional file 1). Animals not assigned to raxi treatment concurrently received a similar volume of a human inactive, non-specific iso-matched IgG1 monoclonal antibody (mAb CAT002 lot number: AG-193-73-M4L originally Human Genome Sciences and now GlaxoSmithKline, Rockville, MD, USA) which had been used in our previous study [21].

To ensure that animals studied had similar starting preload; at baseline, all animals had one to three boluses (20 mL/kg) of normal saline as needed until a PCWP of ≥10 mmHg was achieved. Thereafter, animals receiving HS were administered a single bolus of 20 mL/kg normal saline if the PCWP (note times above) was found to be <10 mmHg. Additionally, if at any time, the mean arterial blood pressure (MAP) decreased to <80 mmHg for >5 min, norepinephrine infusion was initiated at 0.2 μg/kg/min and, if necessary, increased in a stepwise fashion to 0.6, 1, or a maximum of 2 μg/kg/min, every 5 minutes (and similarly titrated down if MAP was >100 mmHg for >5 min). Animals in the no treatment group in Study 1 had measures performed but did not receive titrated fluid or norepinephrine. Technicians blinded to raxi allocation administered all supportive therapies.

Ventilator management, temperature control, and sedation with midazolam, fentanyl, and medetomidine were managed uniformly for all groups based on previously reported protocols [26]. Stepwise ventilator adjustments were made to FiO_2_, positive end-expiratory pressure, and respiratory rate based on continuous pulse oximetry and regularly performed arterial blood gases. All animals received maintenance fluids (Normosol-M at 3 mL/kg/h for the first 36 h, 2 mL/kg/h for the next 36 h, and then 1 mL/kg/h until study completion) [26]. Additional care for all animals included pharmacologic prophylaxis for gastrointestinal stress ulcers (famotidine) and deep venous thrombosis (heparin subcutaneously) and ceftriaxone to prevent catheter-related infections [26].

### Statistical methods

Survival times between the two treatment groups were compared using exact log-rank tests (StatXact, Cytel Software Corp., Cambridge, MA). For all other variables, the change-from-baseline values for individual animals were compared unless there was no baseline value collected. To evaluate shock reversal, we standardized MAP and norepinephrine using Z-scores and then calculated a ‘shock reversal’ score (designated shock index) based on the difference of the MAP Z-score and norepinephrine Z-score, with a higher score indicating improved hemodynamics. Linear mixed models (SAS PROC Mixed, SAS version 9.3, Cary, NC) were used to compare the change from baseline values of different treatments for each time point. All animals were analyzed in the model, and the correlation of animals within each week was accounted for in the model as a random effect. Standard residual diagnostics were used to check model assumptions. Two-sided *p* values of 0.05 or less were considered significant without adjusting for multiple comparisons.

## Results

### Study 1: Effect of hemodynamic support alone or together with PA-mAb during challenge with edema toxin

#### Survival

A total of 40 animals were challenged with 24 h ET infusions in ten weekly experiments (four animals per experiment). Over these experiments, all ten animals receiving no treatment (maintenance fluid only) and all ten animals receiving HS alone (see Methods) died with median survival times (ranges) of 45.2 h (21.0 to 57.1) and 43.8 h (16.8 to 80.3), respectively (*p* = 0.61) (Figure 1). In experiments 1 to 6, six of seven animals receiving HS and raxi administered at 0 h (HS + mAbT0) survived while only one of five animals receiving HS and raxi administered at 12 h (HS + mAbT12) survived, with the median survival times of 96.0 h (39.5 to 96.0) and 55.3 h (12.6 to 96.0), respectively. Compared to HS alone in experiments 1 to 6, HS + mAbT0 improved survival significantly (*p* = 0.02) but HS + mAbT12 did not (*p* = 0.19). To conserve animals but still explore the potential benefit of delayed raxi treatment (<12 h after toxin infusion), in experiments 7 to 10, animals were randomized each week to no treatment (*n* = 1), HS alone (*n* = 1), or HS with raxi administered 6 h after the start of ET (HS + mAbT6, *n* = 2). In these experiments, seven of eight animals receiving HS + mAbT6 survived with a median survival time of 96.0 h (89.5 to 96.0), and this was significantly better than HS alone (*p* = 0.01).

#### Other laboratory data

**Figure 2:**
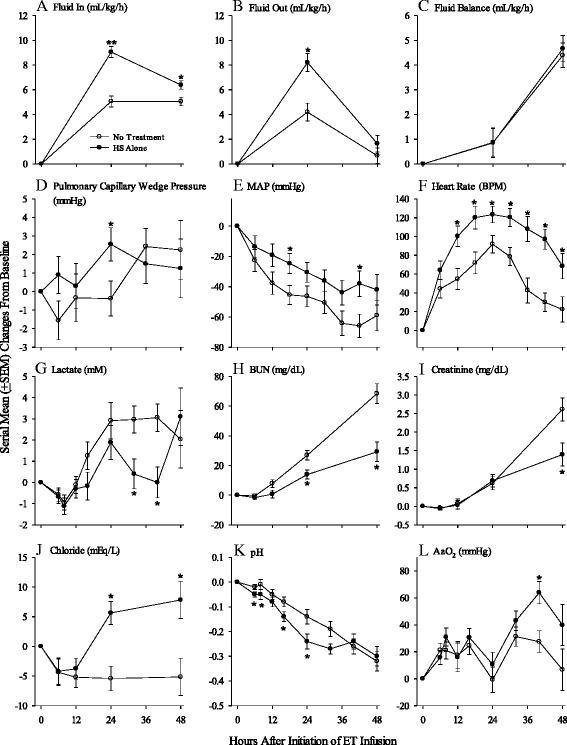
**Serial mean changes for HS animals vs. controls.** This figure compares the serial mean (±SEM) changes from baseline in fluid in **(A)**, fluid out **(B)**, net fluid **(C)**, pulmonary capillary wedge pressure (PCWP) **(D)**, mean arterial blood pressure (**E**; MAP), heart rate (**F**; HR), lactate **(G)**, blood urea nitrogen (**H**; BUN), creatinine **(I)**, chloride **(J)**, pH **(K)**, and alveolar to arterial oxygen gradient (**L**; AaO2) for animals challenged with edema toxin and receiving no treatment (maintenance fluid only) versus hemodynamic support alone (HS; fluids and norepinephrine titrated to pulmonary artery wedge pressure and mean arterial blood pressure, respectively) in Study 1. Units of measure are shown in each panel. *p ≤ 0.05, **p < 0.0001.

**Figure 3:**
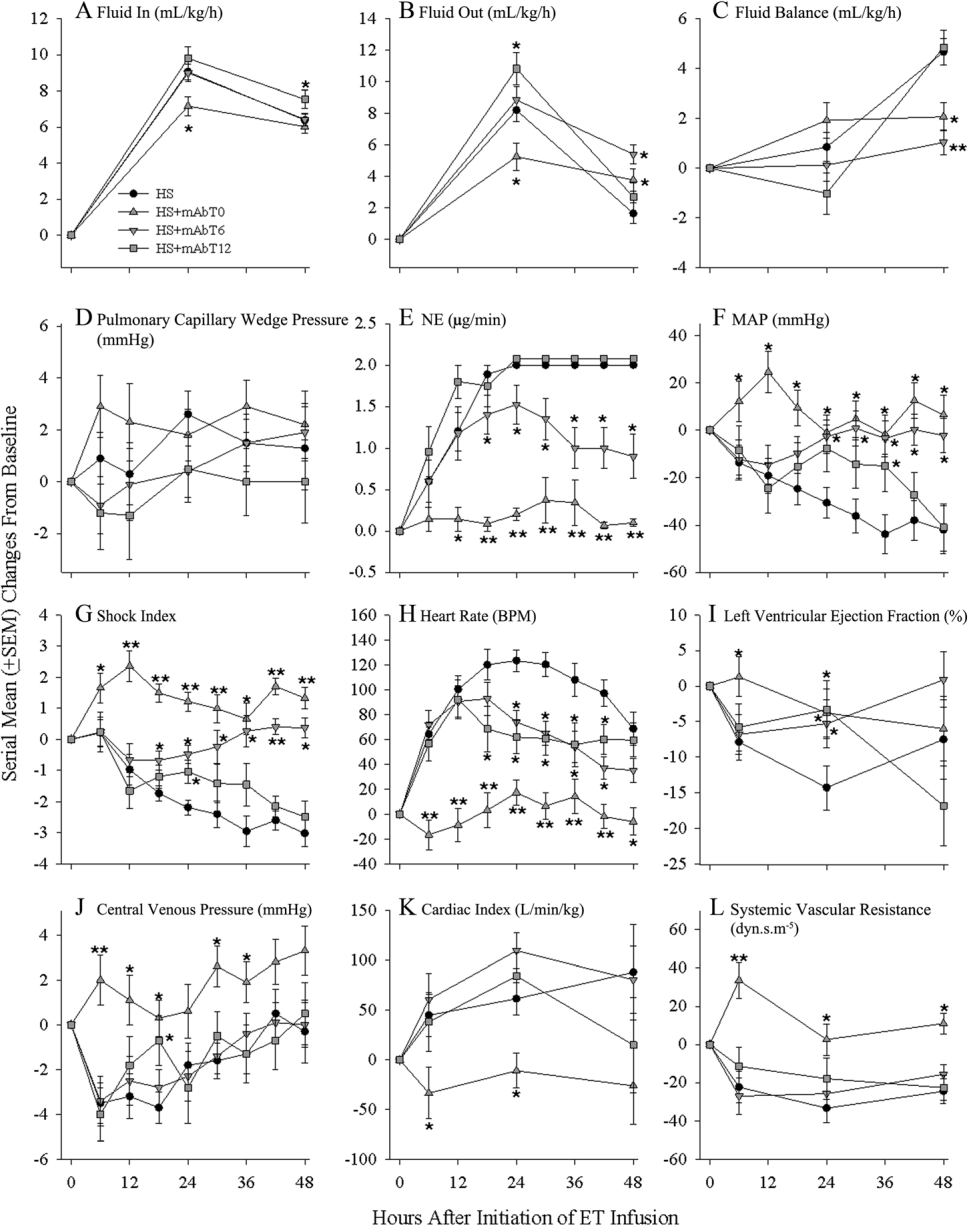
**Serial mean changes in fluids and hemodynamics for HS. vs. raxibacumab animals in study 1.** This figure compares the serial mean (±SEM) changes from baseline in fluid in **(A)**, fluid out **(B)**, net fluid **(C)**, pulmonary capillary wedge pressure (PCWP) **(D)**, norepinephrine use (NE) **(E)**, mean arterial blood pressure (MAP) **(F)**, shock index score **(G)**, heart rate (HR) **(H)**, left ventricular ejection fraction (LVEF) **(I)**, central venous pressure (CVP) **(J)**, cardiac index (CI) **(K)**, and systemic vascular resistance (SVR) **(L)** for animals receiving hemodynamic support alone (HS, fluids and norepinephrine titrated to pulmonary artery wedge pressure and mean arterial blood pressure, respectively) versus HS combined with raxibacumab administered at the start of (HS + mAbT0) or 6 h (HS + mAbT6) or 12 h (HS + mAbT12) after the start of edema toxin challenge in Study 1. Units of measure are shown in each panel (except for shock index score). *p ≤ 0.05, **p < 0.0001.

**Figure 4:**
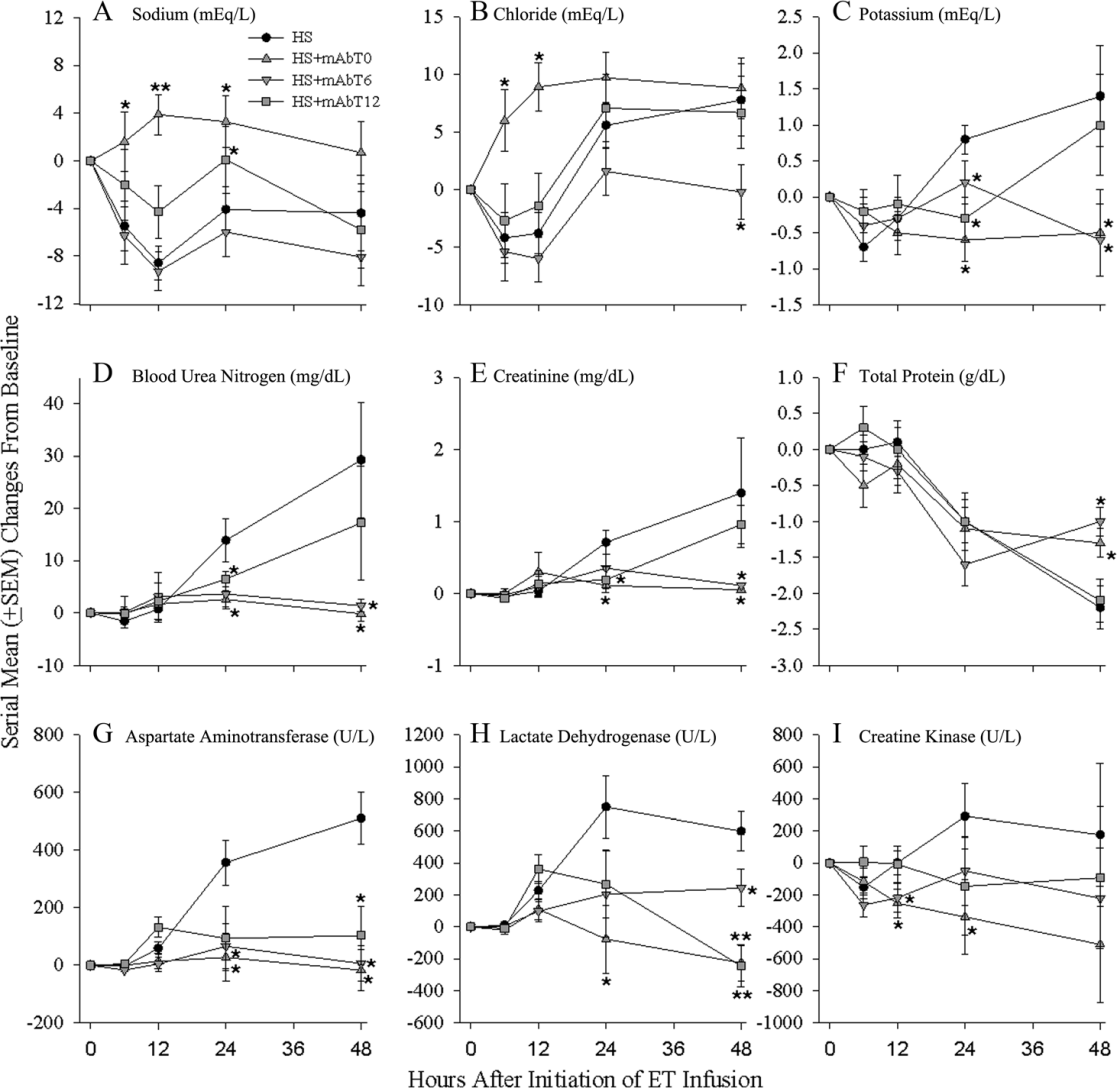
**Serial mean changes in electrolytes for HS. vs. raxibacumab animals in study 1.** This figure compares the serial mean (±SEM) changes from baseline in sodium **(A)**, chloride **(B)**, potassium **(C)**, blood urea nitrogen (BUN) **(D)**, creatinine **(E)**, total protein (TP) **(F)**, aspartate aminotransferase (AST) **(G)**, lactate dehydrogenase (LDH) **(H)**, and creatine kinase (CK) **(I)** for animals receiving hemodynamic support alone (HS, fluids and norepinephrine titrated to pulmonary artery wedge pressure and mean arterial blood pressure, respectively) versus HS combined with raxibacumab administered at the start of (HS + mAbT0) or 6 h (HS + mAbT6) or 12 h (HS + mAbT12) after the start of edema toxin challenge in Study 1. Units of measure are shown in each panel *p ≤ 0.05, **p < 0.0001.

**Figure 5:**
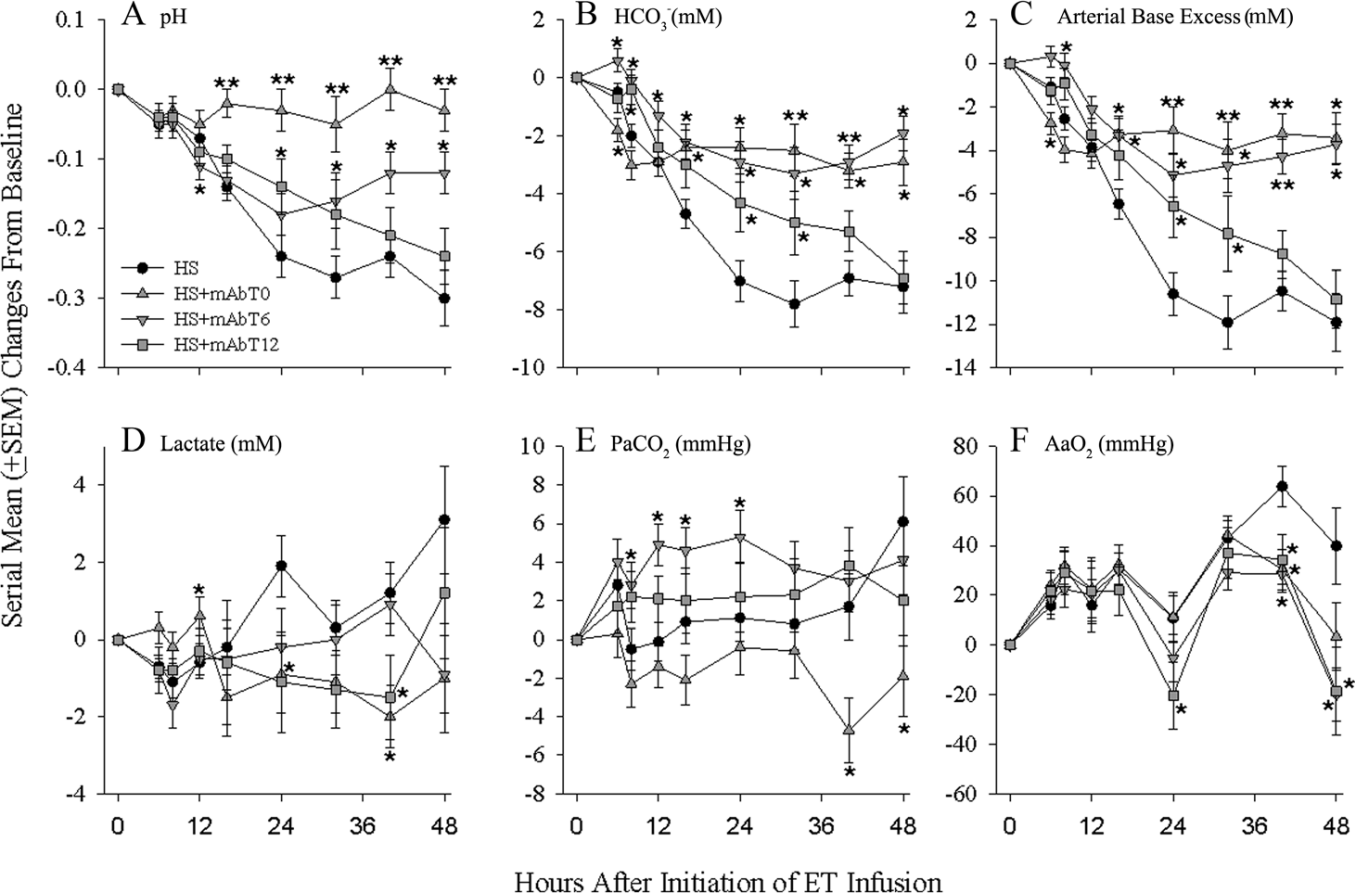
**Serial mean changes in ABG data for HS. vs. raxibacumab animals in study 1.** This figure compares the serial mean (±SEM) changes from baseline in pH **(A)**, HCO_3_^−^ **(B)**, arterial base excess (ABE) **(C)**, lactate **(D)**, PaCO_2_ **(E)**, and alveolar to arterial oxygen gradient (AaO_2_) **(F)** for animals receiving hemodynamic support alone (HS, fluids and norepinephrine titrated to pulmonary artery wedge pressure and mean arterial blood pressure, respectively) versus HS combined with raxibacumab administered at the start of (HS + mAbT0) or 6 h (HS + mAbT6) or 12 h (HS + mAbT12) after the start of edema toxin challenge in Study 1. Units of measure are shown in each panel *p ≤ 0.05, **p < 0.0001.

Since few animals receiving no treatment or HS alone were alive for comparison after 48 h from Study 1 (Figure 1), analysis was conducted over the initial 48 h as lethality developed. To first determine whether there was any evidence of benefit with HS alone, this group was compared to the no treatment group (Figure 2). Then, the effects of HS with raxi administered at each of the three treatment times were compared to HS alone (Figures 3, 4, and 5).

Compared to no treatment, HS animals had increased fluid intake at 24 and 48 h and increased fluid (urine) output at 24 h (all *p* ≤ 0.05), but no significant difference in net fluid balance (Figure 2). Norepinephrine was administered to HS animals by 6 h and then increased to the maximum level by 24 h where it remained until death in all animals. Compared to no treatment, HS significantly increased PCWP at 24 h, MAP at 18, 30, and 42 h, and (possibly related to the maximum norepinephrine dose HS animals received) HR at 12 to 48 h (*p* ≤ 0.05) (Figure 2). Consistent with treatment and improved hemodynamics, HS decreased lactate at 32 and 40 h, blood urea nitrogen (BUN) levels at 24 and 48 h, and creatinine (Cr) at 48 h. HS also increased chloride (Cl^−^) at 24 and 48 h and sodium (Na^+^) at 24 h (*p* ≤ 0.05, data for Na^+^ not shown). Despite decreases in lactate, HS decreased arterial base excess (ABE) levels at 6 to 24 h, pH at 6 to 24 h (except 12 h), and bicarbonate (HCO3^−^) at 8 to 16 h, possibly due to the normal saline administered (*p* ≤ 0.05, ABE and HCO3^−^ not shown). Notably, HS significantly decreased arterial oxygen (PaO_2_) and increased alveolar to arterial oxygen gradient (AaO_2_) at 40 h (*p* ≤ 0.05, data for PaO_2_ not shown). As shown in Figure 2, the significant effects of HS on MAP, lactate, BUN, and Cr resulted in changes closer to baseline values than in controls not receiving treatment.

We next compared the effect of HS combined with raxi versus HS alone (Figures 3, 4, and 5). Since HS + mAbT0 and HS + mAbT6 both significantly increased survival compared to HS, while HS + mAbT12 did not, data are first presented for the two earlier raxi treatment groups. Compared to HS alone, HS + mAbT0 and HS + mAbT6 increased fluid (urine) output and net negative fluid balances at 48 h (*p* ≤ 0.05) (Figure 3). Pulmonary capillary wedge pressure did not differ significantly between groups. Treatment with HS + mAbT0 and HS + mAbT6 decreased norepinephrine requirement, increased MAP and shock index scores, and decreased HR at multiple time points between 12 and 48 h (*p* ≤ 0.05), although these effects occurred earlier and were more pronounced with HS + mAbT0. Both HS + mAbT0 and HS + mAbT6 increased left ventricular ejection fraction (LVEF) at 24 h, total protein and albumin at 48 h, and decreased BUN, Cr, potassium (K^+^), aspartate amino-transaminase (AST), and lactate dehydrogenase (LDH) at 24 and/or 48 h and creatine kinase (CK) at 12 h (*p* ≤ 0.05 for all) (Figures 3 and 4, data for albumin not shown). While HS + mAbT0 and HS + mAbT6 had variable effects early on ABE, pH, and HCO3^−^, both increased these significantly at multiple time points from 12 to 48 h (*p* ≤ 0.05) (Figure 5). Both treatments also increased PaO_2_ and decreased AaO_2_ at either 40 or 48 h (*p* ≤ 0.05, data for PaO_2_ not shown). Compared to HS alone, HS + mAbT0 also decreased fluid intake and output at 24 h, increased CVP from 6 to 18 h and 30 to 36 h, decreased cardiac index (CI) and increased systemic vascular resistance (SVR) from 6 to 24 or 48 h, increased Na^+^ at 6 to 24 h and Cl^−^ at 6 to 12 h, decreased arterial carbon dioxide (PaCO_2_) at 40 to 48 h and first increased lactate at 12 h and then decreased it at 24 and 40 h (*p* ≤ 0.05). Compared to HS alone, HS + mAbT6 decreased Cl^−^ at 48 h and increased PaCO_2_ at 8 to 24 h.

Compared to HS alone, HS + mAbT12 increased fluid intake at 48 h and output at 24 h (both *p* ≤ 0.05), but did not alter net fluid balance. Animals receiving HS + mAbT12 required increasing doses of norepinephrine just like HS alone animals and had modest increases in MAP (significant at 36 h, *p* = 0.05), although shock index scores did not differ significantly. Moreover, HS + mAbT12 treatment was associated with decreased HR at 18 to 42 h, increased HCO3^−^ at 8, 24, and 32 h and ABE at 24 and 32 h, increased PaO_2_ at 48 h and decreased AaO_2_ at 24, 40, and 48 h (all *p* ≤ 0.05, data for PaO_2_ not shown).

As shown in Figures 3, 4, and 5, the effects of either HS + mAbT0, HS + mAbT6, or HS + mAbT12 that were significant on norepinephrine requirements, MAP, Shock Index, HR, LVEF, CVP, CI, SVR, Na^+^, K^+^, BUN, Cr, protein, ASL, lactate, CK, pH, HCO_3_, ABE, lactate, or AaO_2_ resulted in changes closer to baseline values than in animals receiving HS alone.

### Study 2: Effect of hemodynamic support alone or together with PA-mAb during challenge with lethal and edema toxin together

#### Survival

**Figure 6:**
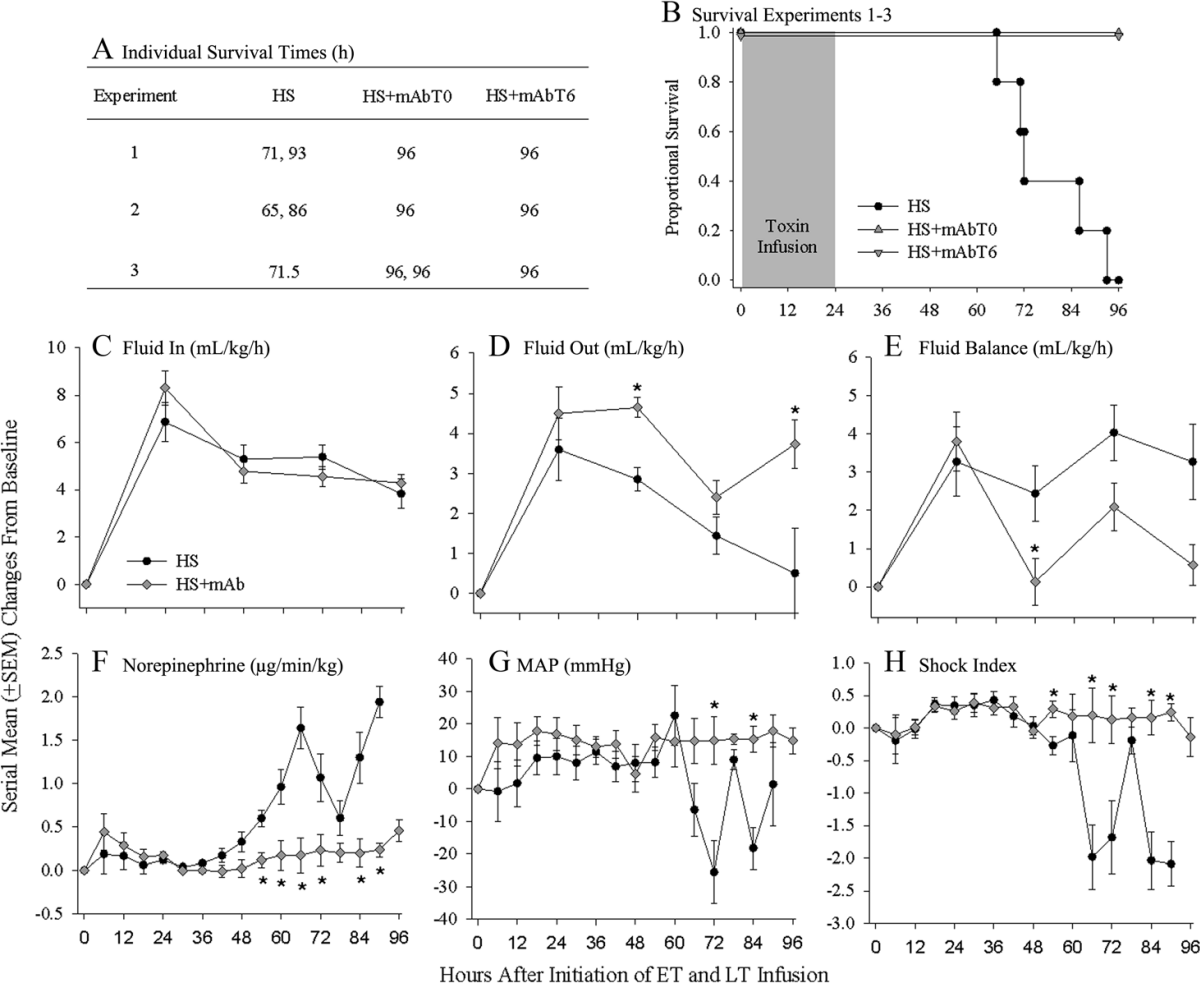
**Survival times and the proportion of animals surviving over time and serial mean changes in parameters in study 2.** This figure shows the individual survival times for each of the animals assigned to hemodynamic support alone (HS; fluids and norepinephrine titrated to pulmonary artery wedge pressure and mean arterial blood pressure, respectively) and HS combined with raxibacumab administered at the start of (HS + mAbT0) or 6 h after (HS + mAbT6) after the start of lethal and edema toxin infusion in each of the three weekly experiments comprising Study 2 **(A)**. This figure also shows the proportion of animals surviving over time in these three experiments combined **(B)**. Finally, this figure compares the serial mean (±SEM) changes from baseline in fluid in **(A)**, fluid out **(B)**, net fluid **(C)**, norepinephrine requirement (NE) **(D)**, mean arterial blood pressure (MAP) **(E)**, and shock index score **(F)**, for animals receiving hemodynamic support alone (HS, fluids and norepinephrine titrated to pulmonary artery wedge pressure and mean arterial blood pressure, respectively) versus combined with protective antigen directed monoclonal antibody administered at the start of (HS + mAbT0) or 6 h (HS + mAbT6) after the start of lethal and edema toxin challenge in Study-2. In panels C-H, the two raxibacumab groups are combined and designated HS+mAb. *p ≤ 0.05, **p < 0.0001.

Twelve animals were challenged with 24 h infusions of LT and ET together in three weekly experiments (four animals per experiment). Five animals were treated with HS alone, and four and three were treated with HS combined with raxi administered at 0 or 6 h, respectively (Figure 6A). While all animals receiving HS alone died with a median survival time (range) of 71.5 h (65 to 93 h), all animals receiving HS with raxi at either 0 or 6 h survived for 96 h (Figure 6A and B). Since these effects on survival were very similar to those observed with HS with raxi treatment during challenge with ET or LT alone (see above for ET and reference 21 for LT), further experiments were not conducted and data from animals receiving raxi at the two treatment times were combined in survival and other data analysis. Compared to HS alone, animals receiving HS with raxi at 0 or 6 h (HS + mAbT0/T6) had significantly improved survival (*p* = 0.01).

#### Other laboratory data

Due to longer survival with HS alone in Study 2, other laboratory data were examined over the entire 96 h observation period. Compared to HS alone, HS + mAbT0/T6 significantly increased fluid (urine) output at 48 and 96 h and net negative fluid balance at 48 h, decreased norepinephrine requirements at 54 to 90 h (except 78 h), increased MAP at 72 and 84 h and shock index score at 54 and 66 to 90 h (except 78 h) (Figure 6C to H), decreased HR at 54 to 78 h, and increased CVP at 60 to 72 and 90 h (*p* ≤ 0.05) (Figure 6C to H, HR and CVP data not shown). Compared to HS alone, HS + mAbT0/T6 also increased Cl^−^ at 6, 12, and 24 h and ABE and HCO3^−^ at 56, 64, and 88 h and decreased AaO_2_ at 48, 56, and 64 h and PT at 72 h (*p* ≤ 0.05, data not shown). As shown in Figures 6, the effects of HS + mAbT0 and HS + mAbT6 on norepinephrine requirements, MAP, and Shock Index resulted in changes closer to baseline values than in animals receiving HS alone. Such changes were also the case for ABE, HCO3^−^, AaO_2_, and PT (not shown).

## Discussion

In summary, without any treatment in Study 1, 24 h ET challenge produced 100% lethality. Treatment with HS alone produced smaller decreases in blood pressure, but did not improve survival from ET challenge. However, HS combined with raxi at either 0 or 6 h but not at 12 h, improved survival significantly compared to HS alone. Similarly in Study 2, with LT and ET challenge together, HS combined with raxi at 0 or 6 h increased survival significantly.

Unlike our prior study where HS improved survival from a lethal LT challenge, HS alone did not improve survival in Study 1 from a similarly lethal ET challenge. Several reasons could account for this difference [21]. First, while lethality with ET was evident before challenge was completed (<24 h), with LT the first death didn’t occur until 24 h after challenge. Thus, the rapidity of lethality with ET may have negated any beneficial survival effect with HS. Second, in a rat aortic ring model, treatment with lethal concentrations of ET but not LT inhibited the contractile response of rings to phenylephrine [27]. In this canine model, ET may have similarly directly inhibited the beneficial vasopressor effects of norepinephrine. Finally, treating shock from ET challenge with HS alone produced hypoxemia. Although ET has generally not produced gas exchange abnormalities in animal models, fluids in HS may have aggravated ETs edema producing actions. Such an effect could have worsened oxygenation and negated benefit with HS. Previously, HS did not worsen oxygenation during LT challenge [18,21].

In contrast to HS alone during ET challenge, HS with raxi administered at 0 or 6 h significantly increased survival. This survival benefit was associated with increased urine output and net negative fluid balance, reduced vasopressor requirements, and improved blood pressure, shock index score, oxygenation, and organ function (i.e., reduced BUN, Cr, and AST). Furthermore, HS with raxi at the start of ET challenge reduced fluid intake at 24 h, while increasing CVP and SVR. The basis for these effects of HS combined with raxi is likely related to evidence that ET contributes to shock via at least two mechanisms. First, ET weakens the adherens junctions connecting endothelial cells and promotes extravasation of fluid and solute [28-30]. Inhibition of ET and improved endothelial barrier function would provide a basis for the decreased fluid requirement and net negative fluid balance, increased CVP, and improved oxygenation noted with HS and raxi compared to HS alone. Secondly, ET may also cause direct arterial relaxation and inhibit arterial contractile responses to catecholamines [31]. Inhibition of these ET effects provides a basis for the reduced norepinephrine requirements and increased MAP, shock index, and SVR noted with HS and raxi compared to HS alone.

Although HS with raxi administered at 12 h after starting ET challenge was associated with a small increase in survival, this was not significant. Thus, in contrast to HS with raxi at 6 h, the rapidity of ETs lethal effects may have limited benefit with delayed treatment at 12 h. By contrast, with LT challenge previously in which lethality was not evident until 48 h, treatment with HS and raxi as late as 12 h did significantly improve survival [21]. Notably though, in the present study with ET, HS with raxi at 12 h did produce small increases in blood pressure, reductions in AST, and improved oxygenation.

It is possible that even without HS; raxi might have been beneficial with ET in Study 1. However, in a previous LT challenge study using this same model, raxi lacked any benefit when administered alone after 0 h but was beneficial for up to 12 h when combined with HS [21]. That finding suggested that HS augmented the effects of raxi, possibly by promoting its tissue distribution. Based on these prior results and because patients with *B. anthracis* shock would typically receive hemodynamic support, we did not test raxi alone in the present study.

In Study 2, HS combined with raxi at 0 or 6 h was also beneficial with LT and ET together, improving urine output and net negative fluid balance and reducing norepinephrine requirements while increasing MAP, shock index score, and survival. Although the number of animals studied was relatively small, the results of Study 2 were very similar to those observed with HS and raxi with either lethal ET challenge in Study 1 or with lethal LT challenge in our prior study [21]. From an animal care and usage standpoint, additional experiments were not appropriate. However, the findings from these three studies together (Studies 1 and 2 here, and a prior LT alone study) provide evidence that agents inhibiting LT and ET may be beneficial when added to conventional hemodynamic support for shock caused by these toxins during *B. anthracis* infection. Such benefit may be twofold, inhibiting organ injury related directly to the toxins and lowering adverse effects of conventional treatments (e.g., fluid overload or vasopressor-induced ischemic injury).

This study has limitations. First, other pathogenic mediators, such as bacterial cell wall and non-toxin proteases, likely contribute to shock with *B. anthracis* infection [4]. Whether hemodynamic support and raxi would have additive benefit during shock related to these other pathogenic mediators is unclear. Second, hemodynamic support in the present study was restricted to fluid and norepinephrine administration. Other vasopressors or inotropes such as phenylephrine, vasopressin, or dobutamine might also have beneficial effects in combination with raxi or other PA directed agents.

## Conclusion

In conclusion, both LT and ET are thought to contribute to shock and organ injury during severe *B. anthracis* infection. Our findings in this canine model suggest that inhibition of these toxins with PA directed monoclonal antibodies such as raxi likely adds to the benefit of conventional treatments.

## Additional file

Additional file 1: Figure S1. This figure shows the time lines of experimental interventions, measurements, and treatments for Study 1 (A) and Study 2 (B). As outlined in ‘Methods’, in Study 1 at the initiation of 24 h edema toxin infusion, animals were randomized to receive hemodynamic support alone, hemodynamic support in combination with protective antigen directed monoclonal antibody (PA-mAb) administered at the time of (0 h) or 6 or 12 h after starting toxin infusion, or no treatment. Hemodynamic support included a single bolus of 20 mL/kg of normal saline if the pulmonary artery occlusion pressure (PAOP, checked every 2 h for the first 8 h and every 4 h thereafter) was <10 mmHg. Also, if at any time mean arterial blood pressure (MAP) decreased to <80 mmHg for >5 min, a norepinephrine infusion was initiated at 0.2 μg/kg/min and, if necessary, increased in a stepwise fashion every 5 min to 0.6 to 1 or a maximum of 2 μg/kg/min. Norepinephrine was titrated down in a step-wise fashion if MAP was greater than 100 mmHg for >5 min. Other abbreviations: HR, heart rate; CVP, central venous pressure; LVEF, left ventricular ejection fraction (measured with echocardiography); CBC, complete blood count; ABG, arterial blood gas. In Study 2, animals were challenged with lethal toxin and edema toxin in combination and were randomized to treatment with hemodynamic support alone or hemodynamic support combined with PA-mAb administered at the time of or 6 h after the start of toxin. Other measurements and treatments were similar to Study 1.
