# Supplementary figures and images for: Raxibacumab augments hemodynamic support and improves outcomes during shock with B. anthracis edema toxin alone or together with lethal toxin in canines
Source: Intensive Care Med Exp. 2015 Feb 28;3:9. doi: 10.1186/s40635-015-0043-4 (PMC4473792; doi:10.1186/s40635-015-0043-4)

Supplemental Figure 1. Study Design

A. Study-1

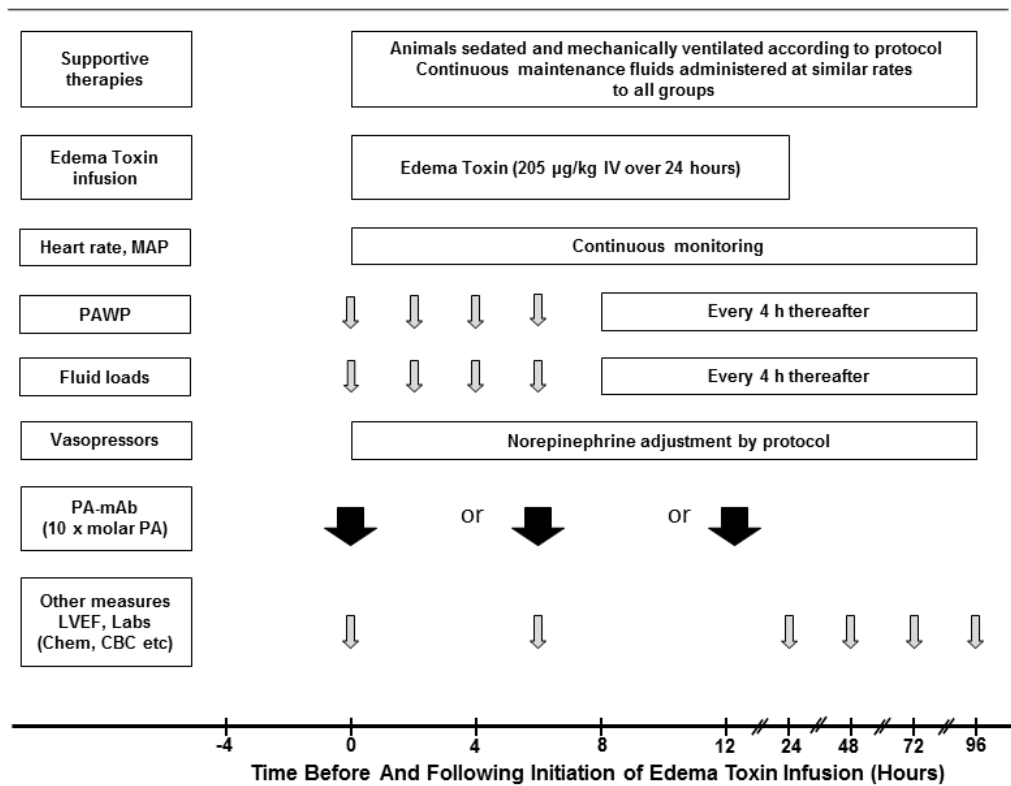

B. Study-2

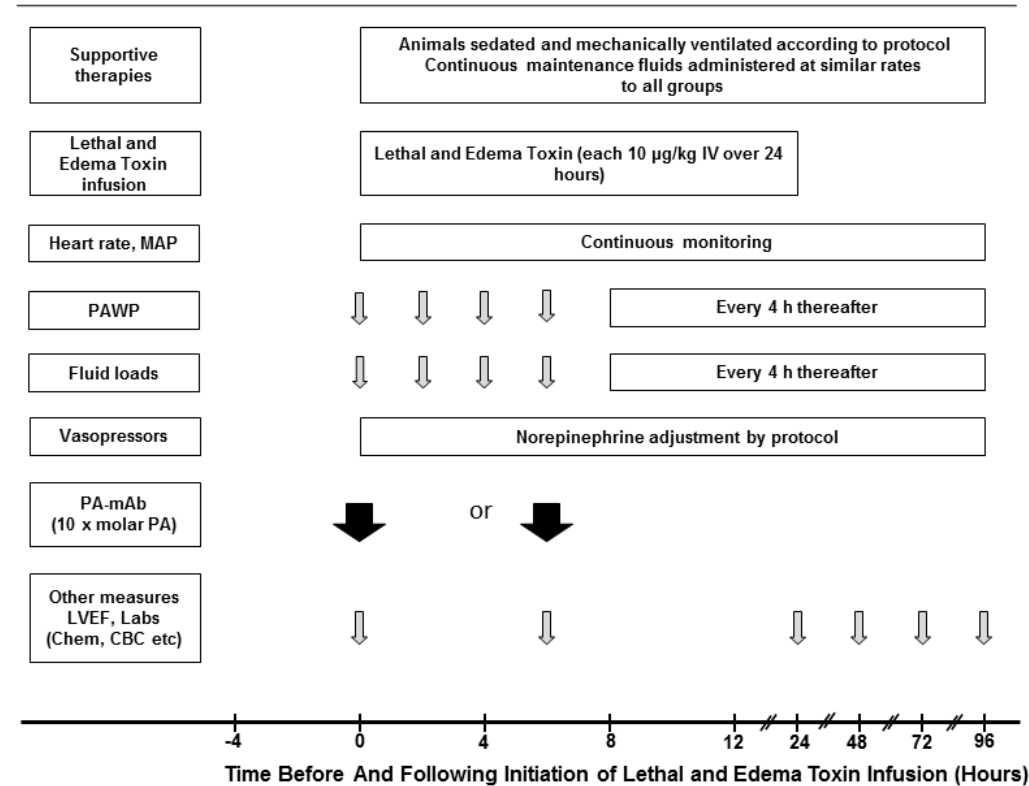

Supplement: Additional file 1: Figure S1. — This figure shows the time lines of experimental interventions, measurements, and treatments for Study 1 (A) and Study 2 (B). As outlined in ‘Methods’, in Study 1 at the initiation of 24 h edema toxin infusion, animals were randomized to receive hemodynamic support alone, hemodynamic support in combination with protective antigen directed monoclonal antibody (PA-mAb) administered at the time of (0 h) or 6 or 12 h after starting toxin infusion, or no treatment. Hemodynamic support included a single bolus of 20 mL/kg of normal saline if the pulmonary artery occlusion pressure (PAOP, checked every 2 h for the first 8 h and every 4 h thereafter) was <10 mmHg. Also, if at any time mean arterial blood pressure (MAP) decreased to <80 mmHg for >5 min, a norepinephrine infusion was initiated at 0.2 μg/kg/min and, if necessary, increased in a stepwise fashion every 5 min to 0.6 to 1 or a maximum of 2 μg/kg/min. Norepinephrine was titrated down in a step-wise fashion if MAP was greater than 100 mmHg for >5 min. Other abbreviations: HR, heart rate; CVP, central venous pressure; LVEF, left ventricular ejection fraction (measured with echocardiography); CBC, complete blood count; ABG, arterial blood gas. In Study 2, animals were challenged with lethal toxin and edema toxin in combination and were randomized to treatment with hemodynamic support alone or hemodynamic support combined with PA-mAb administered at the time of or 6 h after the start of toxin. Other measurements and treatments were similar to Study 1. [file 40635_2015_43_MOESM1_ESM.pdf]
